# Sirtuin activation as a therapeutic approach against inborn errors of metabolism

**DOI:** 10.1007/s10545-016-9939-8

**Published:** 2016-05-04

**Authors:** Jeannette C. Bleeker, Riekelt H. Houtkooper

**Affiliations:** Laboratory Genetic Metabolic Diseases, Academic Medical Center, Meibergdreef 9, 1105 AZ Amsterdam, The Netherlands; Department of Metabolic Diseases, Wilhelmina Children’s Hospital, University Medical Center Utrecht, Lundlaan 6, 3584 EA Utrecht, The Netherlands

## Abstract

Protein acylation has emerged as a large family of post translational modifications in which an acyl group can alter the function of a wide variety of proteins, especially in response to metabolic stress. The acylation state is regulated through reversible acylation/deacylation. Acylation occurs enzymatically or non-enzymatically, and responds to acyl-CoA levels. Deacylation on the other hand is controlled through the NAD^+^-dependent sirtuin proteins. In several inborn errors of metabolism (IEMs), accumulation of acyl-CoAs, due to defects in amino acid and fatty acid metabolic pathways, can lead to hyperacylation of proteins. This can have a direct effect on protein function and might play a role in pathophysiology. In this review we describe several mouse and cell models for IEM that display high levels of lysine acylation. Furthermore, we discuss how sirtuins serve as a promising therapeutic target to restore acylation state and could treat IEMs. In this context we examine several pharmacological sirtuin activators, such as resveratrol, NAD^+^ precursors and PARP and CD38 inhibitors.

## Introduction

Organisms have several mechanisms to regulate cellular processes. In addition to the classical ways of gene regulation such as transcription and translation, post-translational modifications (PTMs) of proteins have emerged as a dynamic mode of regulation, which takes place after proteins are synthesized. Either by covalent bonding or proteolytic cleavage the function and/or structure of a protein can be modified in a more rapid fashion than for instance transcriptional regulation. This allows the cell to respond immediately to environmental changes. Several types of PTM exist, of which phosphorylation is best studied. In recent years, however, protein acylation has emerged as a large family of modifications in which an acyl group — whether an acetyl moiety or a larger acyl group — can be identified on a wide variety of substrate proteins. Protein acylation involves covalent binding of an acyl group to one or more lysine residues of a protein. This neutralizes the positively charged lysine residue, that is often situated in the active or binding site, altering the function or the interaction capabilities of the protein (Walsh et al 2005). At the same time, deacylation enzymes — called sirtuins — have been identified in different subcellular compartments. Sirtuins are active regulators of acylation status and as such control metabolism at many levels (Houtkooper et al 2012).

In mitochondria, PTMs provide a perfect mechanism for quick adaption to changes in energy demand and availability of metabolites. The PTM regulation in this organelle is even tighter considering the fact that intermediary metabolites that are handled by the mitochondrion are substrates for these PTMs as well (Choudhary et al 2014). Indeed, recent work in models for inborn errors of metabolism (IEMs) has marked the extent and variety of protein acylation modifications (Pougovkina et al 2014b; Hirschey and Zhao 2015). In this review, we discuss the pathophysiological role of protein acylation and how sirtuin activation may be a valuable strategy to combat excessive acylation in IEMs.

## Reversible acylation as a post-translational modification

The first discovered type of acylation and therefore best characterized is acetylation, which describes the bond of acetyl-CoA to lysine. Acetylation can affect protein activity, protein-protein interaction, protein stability and subcellular localization of proteins. Functionally, acetylation is well known for activating gene expression (Allfrey et al 1964) by weakening the histone interaction with DNA and trafficking of bromo-domain containing proteins to and from chromatin (Kouzarides 2007; Lee and Workman 2007). It is now evident, however, that acetylation also alters the function of non-histone proteins, such as p53 (Gu and Roeder 1997).

**Fig. 1:**
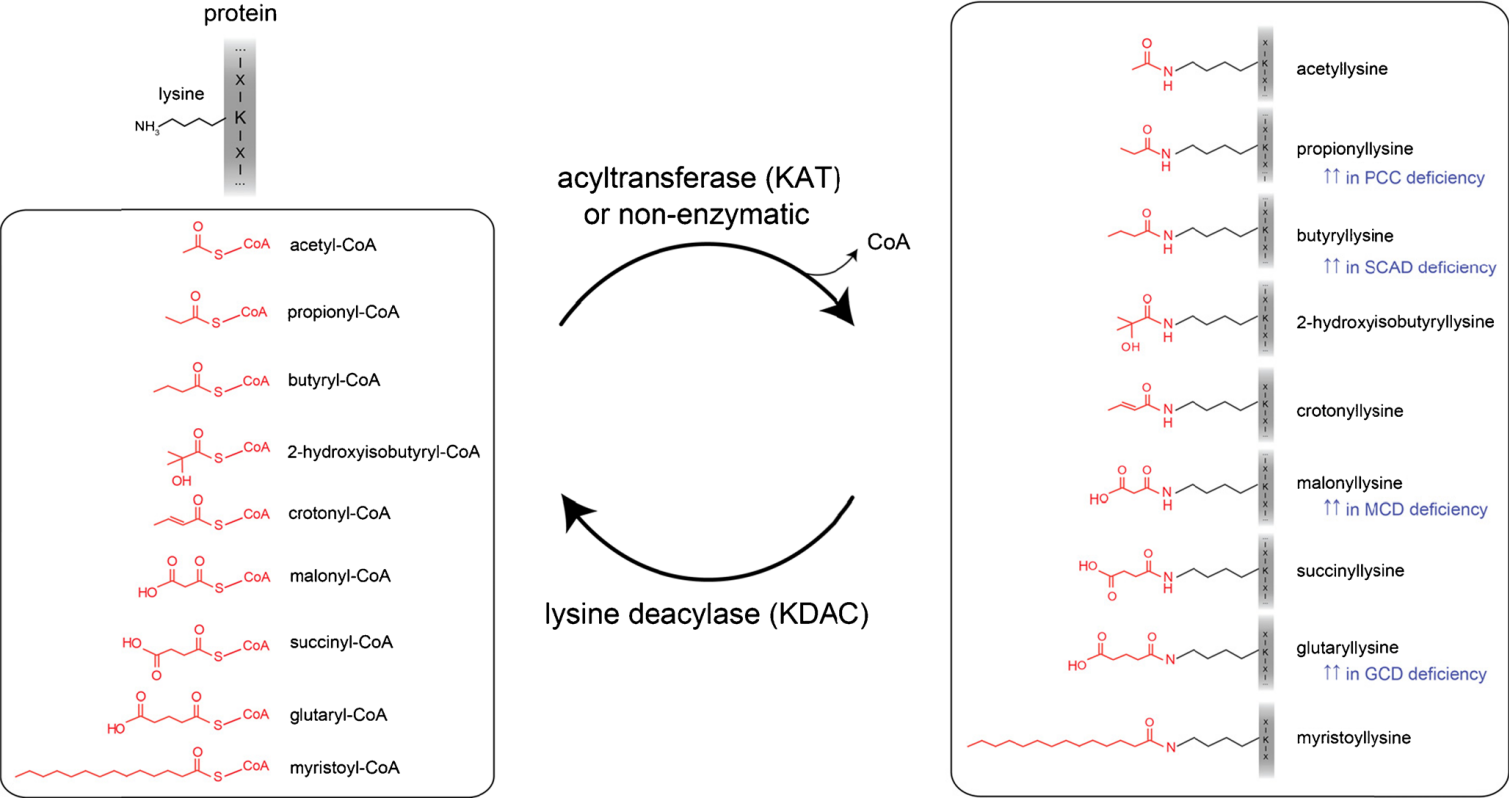
Regulation and chemical structures of lysine acylation modifications, including acetylation, propionylation, butyrylation, 2-hydroxyisobutyrylation, crotonylation, malonylation, succinylation, glutarylation and myristoylation. Lysine acylation is catalyzed by lysine acyltransferase (KAT) and at least partly through non-enzymatic reactions driven by acyl-CoA levels. Lysine deacylation is catalyzed by lysine deacylase (KDAC) enzymes, such as sirtuins. PCC: propionyl-CoA carboxylase; GCDH: glutaryl-CoA dehydrogenase; MCD: malonyl-CoA decarboxylase; SCAD: short-chain acyl-CoA dehydrogenase

Over the past decade it became evident that other short-chain acyl groups can also bind to lysine residues (Lin et al 2012). Lysine propionylation and butyrylation are structurally similar to acetylation (Fig. 1) and occur at many sites that are also acetylated on histones and non-histone proteins, including p53 (Chen et al 2007; Cheng et al 2009; Zhang et al 2009). Lysine malonylation, succinylation and glutarylation are thought to have a more profound impact on protein structure and function since it not only neutralizes the charge of the lysine residue but even charges it negatively (Hirschey and Zhao 2015). Lysine crotonylation and 2-hydroxyisobutyrylation also serve as novel PTMs, but so far only histone targets have been found (Tan et al 2011; Montellier et al 2012; Dai et al 2014).

The spectrum of acylation PTMs was expanded with the identification of regulatory long-chain acylation, in particular lysine myristoylation (Jiang et al 2013). Although this modification was already detected in the 1990s on TNF-α and IL-1α (Stevenson et al 1992; Stevenson et al 1993), it received little attention for many years until a regulatory mechanism was discovered through SIRT6-dependent deacylation (Jiang et al 2013). This member of the sirtuin family, which will be discussed in a later section, preferentially removes long-chain acyl groups from lysine residues, including hexanoyl, octanoyl, decanoyl, dodecanoyl, palmitoyl and oleoyl chains (Feldman et al 2013). Despite the lack of literature on these types of acylation, the discovery of their regulatory mechanisms points at a functional role in PTM.

### Lysine acylation

The (sub)cellular acylation status is dependent on the balance between acylation and deacylation. Acylation can occur either enzymatically or non-enzymatically. In the case of enzymatic acetylation, lysine acetyltransferases (KATs, also referred to as histone acetyltransferases or HATs) transfer an acetyl group from acetyl-CoA to a lysine residue (Roth et al 2001). Although originally reported to acetylate histones only, KATs also acetylate non-histone proteins in different compartments of the cell and members of the p300/CREB family of KATs have been shown to catalyze propionylation, butyrylation, crotonylation and succinylation as well (Chen et al 2007; Cheng et al 2009; Tan et al 2014; Hirschey and Zhao 2015; Sabari et al 2015). In mitochondria, where acetylation is highly abundant (Kim et al 2006), GCN5L1 was reported as a mitochondrial acetyltransferase (Scott et al 2012).

Non-enzymatic acylation of lysine residues is possible under conditions with an alkaline pH and high abundance of substrate (Paik et al 1970; Wagner and Payne 2013). Since the mitochondrion is the only organelle that fits both these criteria, this is considered the principal location for non-enzymatic acylation reactions. Furthermore, acetyl-CoA generated by fatty acid oxidation is a direct substrate for mitochondrial protein acetylation and mitochondrial protein acetylation levels correlate with mitochondrial acetyl-CoA levels (Hirschey et al 2010; Hirschey et al 2011; Pougovkina et al 2014a). Together, these local environmental conditions make it likely that both enzymatic and non-enzymatic acylation occur side by side, at least in mitochondria.

### Sirtuins as lysine deacylase enzymes

Lysine deacetylation or deacylation is catalyzed by lysine deacylases (KDACs, or HDACs in earlier papers) that remove the acyl group from the lysine residue. For an overview of the conventional Zn^2+^-dependent KDACs we refer the reader to earlier comprehensive reviews (de Ruijter et al 2003; Menzies et al 2015). Here, we will focus on the NAD^+^-dependent KDACs — called sirtuins — as they are known for their role in metabolic regulation (Houtkooper et al 2012; Newman et al 2012; Menzies et al 2015). Since sirtuins are NAD^+^ dependent, a physiological or pharmacological increase of NAD^+^ levels leads to sirtuins activation (Houtkooper et al 2010). In mammals, there are seven sirtuins with different subcellular localizations and deacylation targets (Haigis and Sinclair 2010). While sirtuins were originally described as deacetylase enzymes, it has become apparent that longer acyl groups can also be removed, and some sirtuins possess ADP-ribosylation activity (Houtkooper et al 2012). The best-described sirtuin in this context is the mitochondrial SIRT5, which has demalonylation, desuccinylation and deglutarylation activity (Du et al 2011; Peng et al 2011; Park et al 2013; Rardin et al 2013; Tan et al 2014; Nishida et al 2015).

### Metabolic consequences of deacylation dynamics

Deacylation of proteins by sirtuins can transform the protein to its active state, but can also result in an opposite effect, such as protein degradation. For instance, SIRT1, the best-described sirtuin, is an important regulator of glucose and fat metabolism in response to energetic challenges (Houtkooper et al 2012). During energy limitation, elevated NAD^+^ levels induce SIRT1, which activates several proteins that regulate the switch from glucose metabolism to fat oxidation, such as FOXO1, PPARα and PPARγ coactivator-1 α (PGC-1α). Both in liver and skeletal muscle, this activation leads to inhibition of glycolysis and enhanced fatty acid oxidation (Purushotham et al 2009; Philp et al 2011). On the other hand, SIRT1 can also inactivate proteins by deacetylation. It represses for example the transcription of uncoupling protein 2 (UCP2) in the pancreas, resulting in increased insulin secretion (Moynihan et al 2005; Bordone et al 2006). Similarly, SIRT1 activation leads to degradation of CREB-regulated transcription cofactor 2 (CRTC2) that suppresses the transcription of gluconeogenic genes (Liu et al 2008).

SIRT3 is the principal regulator of mitochondrial deacetylation and is highly expressed in liver, kidney and heart (Ahn et al 2008). It targets several metabolic enzymes, including long-chain acyl-CoA dehydrogenase (LCAD), glutamate dehydrogenase (GDH) and 3-hydroxy-3-methylglutaryl CoA synthase 2 (HMGCS2), which are involved in fatty acid oxidation, ketone body production and TCA cycle. SIRT3 therefore is essential in the switch from glucose metabolism to lipid and amino acid catabolism, that is necessary to adapt to fasting (Hebert et al 2013). Moreover, SIRT3 also activates urea cycle enzyme ornithine transcarbamoylase and protects the cell from reactive oxygen species (ROS) via activation of superoxide dismutase 2 (SOD2) (Chen et al 2011; Hallows et al 2011).

In addition to direct regulation, acetylation also plays a part in PTM crosstalk, where acetylation either blocks a residue so that another PTM cannot bind, or affects binding of nearby PTMs. For example, acetylation of sterol regulatory element-binding protein 1 (SREBP1a), that controls lipogenesis, blocks the binding of ubiquitin and stabilizes the protein (Giandomenico et al 2003). Conversely, acetylation of phosphoenolpyruvate carboxykinase (PEPCK) stimulates interaction with E3 ubiquitin ligase, promoting degradation of the protein (Jiang et al 2011). Altogether, the central position of sirtuin proteins allows these proteins to integrate metabolic cues into pleiotropic adaptive responses.

## Acylation in inborn errors of metabolism

In several inborn errors of metabolism (IEMs), defects in amino acid and fatty acid metabolic pathways can cause accumulation of acyl-CoAs in different compartments of the cell. It seems reasonable that high abundance of substrate can have a direct effect on lysine acylation and the regulation of proteins, especially in mitochondria, where a pH of around 8 facilitates non-enzymatic reactions of acyl-CoA with lysine residues of proteins (Wagner and Payne 2013). Indeed, several mouse or cell models for IEM, in which acyl-CoAs accumulate, display high levels of lysine acylation, including models for propionyl-CoA carboxylase (PCC) deficiency (OMIM 606054), glutaryl-CoA dehydrogenase (GCDH) deficiency (OMIM 231670), malonyl-CoA decarboxylase (MCD) deficiency (OMIM 248360) and short-chain acyl-CoA dehydrogenase (SCAD) deficiency (OMIM 201470) (Pougovkina et al 2014b; Tan et al 2014; Colak et al 2015).

In PCC deficient patients the conversion of propionyl-CoA to methylmalonyl-CoA is compromised. Clinically, patients may present with a variety of symptoms, including seizures, encephalopathy, intellectual disability and cardiomyopathy. Nowadays, many countries have included PCC deficiency in their newborn screening programs, but before introduction of these screening programs patients would be diagnosed in the neonatal period because of rapidly progressing symptoms or at a later stage in childhood with a more chronic form of the disease (Mardach et al 2005; Fenton et al 2011). Biochemically, PCC deficiency results in metabolic acidosis, ketotic hyperglycinemia, hypoglycemia and elevated levels of propionic acid and propionyl carnitine in plasma and urine (Schwab et al 2006; de Keyzer et al 2009; Fenton et al 2011). One of the possible pathophysiological mechanisms in PCC deficiency might be the inhibitory effect of propionyl-CoA on pyruvate dehydrogenase (PDG), α-ketoglutarate dehydrogenase (KGDH), OXPHOS complex III and succinate-CoA ligase (Stumpf et al 1980; Schwab et al 2006). Recently, an increase in lysine propionylation was discovered in patients with PCC deficiency (Pougovkina et al 2014b), which could explain how propionyl-CoA mechanistically inhibits these enzymes.

GCDH deficiency results in impaired breakdown of lysine, hydroxylysine and tryptophan. Clinically, patients often present with macrocephaly at birth and develop neurological symptoms (e.g. dystonia, dyskinesia and ataxia) shortly after (Hedlund et al 2006). Like PCC deficiency, GCDH deficiency is in many countries included in the newborn screening program. Biochemically, GCDH deficiency results in elevated levels of glutaric acid in plasma and urine. In GCDH deficient mice, the accumulation of glutaryl-CoA has been shown to enhance lysine glutarylation with subsequent inhibition of carbamoyl phosphate synthase 1 (CPS1) (Tan et al 2014).

In MCD deficiency the conversion of malonyl-CoA to acetyl-CoA and carbon dioxide is impaired. Patients may present with a variety of clinical symptoms including developmental delay, muscle weakness, seizures and cardiomyopathy (Salomons et al 2007). Biochemically, MCD deficiency results in metabolic acidosis, hypoglycemia and elevated levels of malonic and methylmalonic acid in urine and malonylcarnitine in plasma. In fibroblasts of MCD deficient patients, the accumulation of malonyl-CoA is associated with increased lysine malonylation (Pougovkina et al 2014b). Recent proteomic analysis of MCD deficient mouse liver and fibroblasts of MCD deficient patients identified lysine malonylated sites at many proteins located in mitochondria, cytosol and nucleus (Colak et al 2015). Furthermore, the malonylated proteins in MCD deficient fibroblasts were also involved in fatty acid oxidation, including very long chain acyl-CoA dehydrogenase (VLCAD) and long-chain 3-hydroxyacyl-CoA dehydrogenase (LCHAD). The higher degree of VLCAD malonylation is associated with decreased VLCAD enzyme activity in MCD human fibroblasts. In addition, mitochondrial oxygen consumption was decreased in MCD deficient fibroblasts, particularly when fatty acids were used as a substrate (Colak et al 2015). Interestingly, pathophysiological mechanisms for the cardiomyopathy in MCD deficiency and fatty acid oxidation disorders are poorly understood, but these recent findings might direct to a common pathogenic origin.

SCAD deficiency results in elevated levels of butyryl CoA, butyric acid and butyrylcarnitine. SCAD deficiency is considered clinically irrelevant, since most ‘patients’ stay asymptomatic (van Maldegem et al 2010), but SCAD deficient fibroblasts also serve as a cell model in which increased levels of lysine butyrylation can be studied (Pougovkina et al 2014b).

Finally, increased lysine acetylation has also been demonstrated in mouse models for IEM in the absence of primary acetyl-CoA accumulation. The altered mitochondrial redox state in mouse models for frataxin deficiency (OMIM 229300) and mitochondrially encoded cytochrome c oxidase subunit 1 (MT-CO1) deficiency (OMIM 220110) inhibits deacetylation by SIRT3 leading to hyperacetylation (Wagner et al 2012). It is not unlikely that this mechanism could be applied to other respiratory chain defects as well.

Since the extent of lysine acylation is only starting to emerge, particularly through the work in these models of IEM, the metabolic consequences of lysine acylation require further elucidation. This PTM might turn out to contribute substantially to the pathophysiology in metabolic dysregulation, although it cannot be excluded that specific lysine acylation modifications may represent adaptive responses with profitable outcome.

## Pharmacological sirtuin activation to treat inborn errors of metabolism

**Fig. 2:**
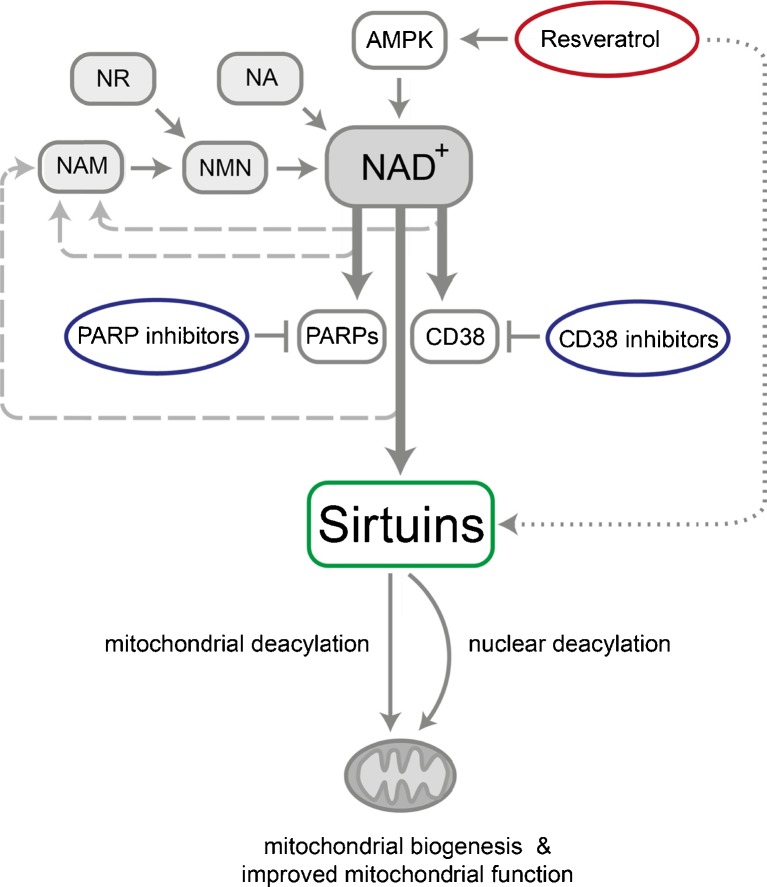
Pharmacological activation of sirtuins. Sirtuins can be activated in multiple ways. Resveratrol activates sirtuins, although the mechanism is still debated. Two proposed modes of activation include (1) activation of AMPK; (2) direct activation. Sirtuins can also be activated through increasing the levels of its substrate NAD^+^. This can be achieved through (**a**) boosting NAD^+^ synthesis from precursors nicotinic acid (NA), nicotinamide riboside (NR) or nicotinamide mononucleotide (NMN); (**b**) inhibiting the activity of major NAD^+^ consuming pathways, such as poly(ADP-ribose) polymerases (PARPs) or the cyclic ADP-ribose synthase CD38. Activating sirtuins can improve the acylation state at various levels and mitochondrial function

Considering that aberrant protein acylation functionally impairs the activity of various enzymes in inborn errors of metabolism, removal of acyl groups from lysine residues by sirtuins can rescue the protein to its native state that is accessible to other PTMs. Therefore boosting the deacylase activity of sirtuins could be an interesting therapeutic approach (Houtkooper and Auwerx 2012). In addition to this direct effect on acylation, SIRT1 activation also leads to enhanced mitochondrial biogenesis and could thereby increase the residual activity of the enzyme that is defective in a certain IEM. Several of such approaches have emerged from the work on common metabolic diseases, which could serve as a framework for translation to the field of inborn errors (Fig. 2).

The most widely used sirtuin activator is the polyphenol resveratrol. Resveratrol, which can be found in grape skin, was originally identified as a SIRT1 activator that extends lifespan in yeast and several other organisms (Howitz et al 2003; Bauer et al 2004; Wood et al 2004; Viswanathan et al 2005; Baur et al 2006; Valenzano et al 2006; Pearson et al 2008; Rascon et al 2012; Yu and Li 2012; Strong et al 2013; Wang et al 2013). Following this initial success, numerous small molecule mimics were identified that induce sirtuin activity (Feige et al 2008; Smith et al 2009; Hoffmann et al 2013). Although the exact molecular mechanism of resveratrol and the other sirtuin activators has become a matter of debate (Bitterman and Chung 2015), it appears uncontested that resveratrol activates SIRT1 and induces its downstream pathways. Indeed, in various models resveratrol induces mitochondrial biogenesis via the coactivator PGC-1α (Rodgers et al 2005). As such, resveratrol, but also the other sirtuin activators, improves metabolic homeostasis in mice, particularly in mice fed a high fat diet, for instance by improving glucose sensitivity, cold tolerance and exercise capacity (Baur et al 2006; Lagouge et al 2006; Feige et al 2008; Um et al 2010). In humans, resveratrol also improved glucose sensitivity in type 2 diabetic and healthy obese men (Timmers et al 2011; Bhatt et al 2012; Konings et al 2014), although other studies report no clinical effect of supplementation (Yoshino et al 2012; Poulsen et al 2013). No clinical studies have been performed yet with resveratrol in patients with inborn errors of metabolism, but in vitro studies reported improved metabolic function in fibroblasts of patients with mitochondrial fatty acid oxidation defects (Bastin et al 2011; Aires et al 2014), respiratory chain deficiencies (Lopes Costa et al 2014) and propionic acidemia (Gallego-Villar et al 2014). Although additional work is needed to elucidate the mechanisms underlying this restoration, it is likely due to an increase in residual activity of the defective enzyme, caused by PGC-1α-dependent mitochondrial biogenesis.

A second strategy to activate sirtuins is to increase the production of its substrate NAD^+^. Nicotinic acid (NA, or niacin), nicotinamide (NAM), nicotinamide mononucleotide (NMN) and NAM riboside (NR) are all NAD^+^ precursors that can boost NAD^+^ levels in different tissues (Jackson et al 1995; Bieganowski and Brenner 2004; Belenky et al 2007; Yoshino et al 2011; Canto et al 2012). NA is widely used as treatment for dyslipidemia (Altschul et al 1955), and can activate SIRT1 (Li et al 2015), but at the same time causes flushing as an adverse effect mediated through the membrane receptor GPR109A (Benyo et al 2005). The other NAD^+^ precursors NAM, NR and NMN do not activate GPR109 but still increase NAD^+^ levels and improve metabolic parameters in rodents that were fed with a high fat diet (Yoshino et al 2011; Canto et al 2012; Yang et al 2014). NAM might, however, play a more complex role, since it has also been shown to inhibit deacylation by sirtuins (Bitterman et al 2002; Peng et al 2011). The only clinical result so far comes from the NAD^+^ precursor acipimox, which improves muscle mitochondrial function and glucose homeostasis in type 2 diabetes patients (van de Weijer et al 2015). In the field of IEM, NR was shown to restore mitochondrial homeostasis in fibroblasts from patients with a mitochondrial respiratory chain defect (Felici et al 2015). Furthermore, NR induces mitochondrial biogenesis in skeletal muscle of mice that suffer from mitochondrial myopathy (Khan et al 2014) and induces OXPHOS-related gene expression and improves motor performance in mice that suffer from cytochrome c oxidase deficiency (Cerutti et al 2014).

NAD^+^ levels can also be increased by limiting its catabolism. Two major pathways compete with sirtuins for the utilization of NAD^+^, i.e. poly(ADP-ribose) polymerases (PARPs) and cyclic ADP-ribose synthases, such as CD38 (Houtkooper et al 2010). Inhibition of these latter pathways hence leads to increased NAD^+^ levels that become available to activate sirtuins. In line with this idea, PARP inhibition in mice increases NAD^+^ levels in various tissues, accompanied by enhanced mitochondrial biogenesis, and improved energy expenditure (Bai et al 2011; Cerutti et al 2014; Pirinen et al 2014). Similarly, deletion or inhibition of CD38 reduced global acetylation and improved glucose and lipid homeostasis in mice as well (Barbosa et al 2007; Escande et al 2013).

## Conclusions

While sirtuin activation seems a promising avenue for IEM treatment, the mode of activation may dictate the efficacy and is dependent on the pathophysiology of the disease. Most of the beneficial effects of resveratrol and PARP inhibitors have been attributed to activation of the nuclear SIRT1, either directly or through the accumulation of nuclear NAD^+^, and likely rely on enhanced mitochondrial biogenesis and upregulation of residual activity. NR and other NAD precursors, however, also reach other compartments of the cell, including mitochondria, and lead to a marked activation of SIRT3 and possibly also SIRT5 (Canto et al 2012). As such, this treatment may be better suited to remove acylation PTMs in cells that accumulate acyl-CoAs and show features of hyperacylation. Regardless of the mechanisms, it is evident that the emerging interest and knowledge about acylation as a PTM introduces a new pathophysiological mechanism in IEM with promising opportunities for a new therapeutic approach. More work is needed to better establish the dynamics of sirtuin activation upon treatment, but combined with the pathophysiological mechanisms pertaining to IEMs this will guide the preferred treatment strategy.
